# Indents

**Published:** 1904-06-15

**Authors:** 


### INDENTS.

☞ **Brief Articles of Technical or Scientific Value will receive notice and comment. Contributions invited.**

**After-pain from Extracting** May be prevented by administering one anti-kamnia and codeine tablet before operating and one after.

**Clay Pipe-stems.** Cut clay pipe stems into one-half inch lengths and use them to support pin crowns when baking in the furnace. They will stand the heat of any furnace.—G. B. Speer, in *Summary*.

**Dentists in Germany.** The German Empire has a population of 58,000,000, and they are served by a corps of 2,000 dentists. It would keep an American dentist rather busy caring for a population of 29,000 people.

**Gum Retractor.** Dr. Kimball, of New York, in the May *International*, suggests making a gum retractor of thin sheet steel cut to fit the contour of the tooth and then soldered with soft solder to an old instrument handle.

**Hydrogen Dioxid and Lime Water.** When about to use hydrogen dioxid to prevent its acid reaction, mix it with an equal volume of lime water. It will be equally effective as a disinfectant, but not escharotic in action.

**Adrenalin Solution.** W. J. Cameron, of Des Moines, Iowa, says a few drops of adrenalin solution enables one to stop the hemorrhage when extracting roots buried beneath the gum, so that it becomes necessary to cut it away to get a clear vision.

**Use for Worn Mandrels.** When the screw thread is worn so it will not hold, mount the stone with sealing wax, warmed and pressed home and held until cool. Cotton warmed on the screw thread will also cause it to hold temporarily.—*Review.*

**To Repair Hole in a Crown.** A small button of pure or coin gold hammered over a hole in the drawplate, corresponding in size to the hole in the crown, makes a convenient rivet which can be attached with solder, or if a bridge is involved can be riveted.—Oliver Martin, Ottawa, Can., in *Review.*

**Tri-Chloracetic Acid.** This is an excellent treatment for pyorrhea as it arrests the formation of pus quickly. It acts like a charm in putrescent pulp canals. Carefully applied to spongy gums, it gives better results than anything I have used. It is also excellent in pericementitis arising from calcic deposits. Because of its astringent and escharotic action it destroys abnormal surface tissue and resuscitates it after a single application.—H. C. McK., in *Dental Brief.*

**Sponge Tin as A Filling Material.** Dr. Scheurer, of Bohemia, recommends the use of sponge tin as a workable metal for filling teeth. It is made by precipitating from a solution stannic chloride by means of a strip of pure zinc. It is easily manipulated, much as crystal or mat gold. A cavity can be nearly filled with it, but the occlusal surface must be made with some form of mat gold, to secure a hard surface. No other form of gold will adhere to this sponge tin like the crystal gold. It may be used in inclosed crown cavities, or even in front teeth, if the cavity shall have been previously lined with a layer of number thirty gold foil. The author also uses this sponge tin in combination with cement. The cement powder and pulverized sponge tin are mixed with cement liquid and when inserted in the tooth and has become hard, it polishes with a metallic surface.—May *Cosmos.*

